# Protective Effect of Liposomal Epigallocatechin-Gallate in Experimental Gentamicin-Induced Hepatotoxicity

**DOI:** 10.3390/antiox11020412

**Published:** 2022-02-17

**Authors:** Adriana Elena Bulboacă, Alina Silvia Porfire, Vasile Rus, Cristina Ariadna Nicula, Corneliu Angelo Bulboacă, Sorana D. Bolboacă

**Affiliations:** 1 Department of Pathophysiology, Iuliu Hațieganu University of Medicine and Pharmacy, 400012 Cluj-Napoca, Romania; adriana.bulboaca@umfcluj.ro; 2 Department of Pharmaceutical Technology and Biopharmaceutics, Iuliu Hațieganu University of Medicine and Pharmacy, 400012 Cluj-Napoca, Romania; aporfire@umfcluj.ro; 3 Department of Cell Biology, Histology and Embryology, University of Agricultural Sciences and Veterinary Medicine, 400375 Cluj-Napoca, Romania; vasile.rus@usamv.ro; 4 Department of Ophthalmology, Iuliu Hațieganu University of Medicine and Pharmacy, 400012 Cluj-Napoca, Romania; cristina.nicula@umfcluj.ro; 5 Department of Neurology and Pediatric Neurology, Iuliu Haţieganu University of Medicine and Pharmacy, 400012 Cluj-Napoca, Romania; angelo.bulboaca@umfcluj.ro; 6 Department of Medical Informatics and Biostatistics, Iuliu Hațieganu University of Medicine and Pharmacy, 400349 Cluj-Napoca, Romania

**Keywords:** gentamicin-induced hepatotoxicity, epigallocatechin gallate (EGCG), metalloproteinase (MMP)

## Abstract

Our study aimed to assess the effect of liposomal epigallocatechin-gallate (LEGCG) compared with epigallocatechin-gallate (EGCG) solution on hepatic toxicity induced by gentamicin (G) administration in rats. Five groups were evaluated, a control group (no G administration) and four groups that received G (1 mL, i.p, 80 mg/kg b.w. (body weight/day), for 7 days) to which we associated daily administration 30 min before G of EGCG (G-EGCG, 2.5 mg/0.1 kg b.w.), LEGCG (G-LEGCG, 2.5 mg/0.1 kg b.w.) or silymarin (100 mg/kg b.w./day). The nitro-oxidative stress (NOx), catalase (CAT), TNF-α, transaminases, creatinine, urea, metalloproteinase (MMP) 2 and 9, and liver histopathological changes were evaluated. LEGCG exhibited better efficacy than EGCG, improving the oxidant/antioxidant balance (*p* = 0.0125 for NOx and 0.0032 for CAT), TNF-α (*p* < 0.0001), MMP-2 (*p* < 0.0001), aminotransferases (*p* = 0.0001 for AST and 0.0136 for ALT), creatinine (*p* < 0.0001), urea (*p* = 0.0006) and histopathologic liver changes induced by gentamicin. Our study demonstrated the beneficial effect of EGCG with superior results of the liposomal formulation for hepatoprotection in experimental hepatic toxicity induced by gentamicin.

## 1. Introduction

Gentamicin (G) is an aminoglycoside commonly used in clinical practice. Despite beneficial effects against bacterial infection, gentamicin-induced toxicity is one of the most critical side effects that require precaution when administering the drug. Due to their active participation in various metabolic pathways, the kidney and liver are particularly exposed to damage associated with drug toxicity. Hepatotoxicity and nephrotoxicity are rare effects of gentamicin administration and are primarily associated with overdoses. Hepatotoxicity is considered one of the most important toxic effects due to the persistence of inflammation that triggers hepatic fibrosis, and consequently, hepatic failure [1,2]. Deciphering gentamicin-induced liver-toxicity associated mechanisms can be challenging due to the various and complex molecular mechanisms involved. The active role of the kidney and liver in different metabolic loops particularly exposes these organs to oxidative-stress induced lesions [1]. Gentamicin-induced hepatotoxicity has been previously demonstrated in experimental studies [3,4,5]. Oxidative stress and inflammation are significant pivotal pathogenetic mechanisms in triggering hepatic fibrosis and its progression [6]. The hepatic fibrosis mechanism shares a similar pattern regardless of its cause and the elimination of etiological factors could reverse fibrosis if it is in an incipient phase. Removal of etiological factors can also reverse fibrosis in advanced stages, but the process is slow [6]. Diminishing the life-threatening complications of liver fibrosis can be a critical therapeutically target, even if the fibrotic process is advanced. Therefore, therapeutic interventions are studied to decrease oxidative stress and inflammation and prevent hepatic fibrosis [7].

Special attention has been dedicated to nutraceutical compounds as adjuvant therapies as effective molecules for oxidative stress and inflammation reduction. Protection against oxidative stress injury and, concomitantly, against associated inflammation is the main target of these therapies. Endogenous antioxidant enzymes are essential components of the cellular stress response, playing an important role in removing oxygen reactive species before they can damage target cell’s DNA. Consequently, an ideal therapy should be addressed to both sides of the oxidative stress/antioxidant balance, reducing the amount of reactive oxygen and nitrogen species as part of oxidative/nitro-oxidative stress and, on the other side, intensifying cellular antioxidant activity. Silymarin, a complex mixture of polyphenolic molecules from *Silybum marianum*, is known as a hepatoprotective compound. The plant flavoligants and flavonoid taxifolin reduce free radicals inducing antioxidant effects [8]. It has been suggested that silymarin can contribute to the cytoprotection of the hepatic cells by reducing cyclooxygenase activity and, consequently prostaglandin and leukotriene formation [9]. Silymarin can also enhance the activity of antioxidant enzymes such as hepatic glutathione, contributing to the antioxidant defense mechanisms in the liver tissue [8]. Thus, silymarin mediates the increase of hepatocyte protein synthesis, slowing the calcium metabolism in the hepatic cells, protecting against genomic injury, and reducing hepatic cell destruction, consecutive inflammation and fibrosis [10]. All these properties recommend silymarin as an effective hepatoprotective natural compound.

Various nutraceutical adjuvant therapies have been proposed to diminish the toxic side effects of gentamicin [2,11,12]. Green tea, extensively consumed worldwide, contains numerous molecules with antioxidant effects, so it is commonly used as an antioxidant and anti-inflammatory nutraceutical adjuvant therapy [13]. Catechin compounds, and a particularly major molecule in green tea, epigallocathehin gallat (EGCG), have some of the most potent antioxidant and anti-inflammatory activities [13]. Epigallocatechin gallate (EGCG) is also the most abundant antioxidant and anti-inflammatory molecule in green tea [14], therefore, the research on this molecule’s properties is extensive [15]. Due to these properties, EGCG has demonstrated beneficial effects in various diseases such as diabetes mellitus, obesity, migraine, stroke, Parkinson’s and Alzheimer’s diseases [2,13,16,17]. The mechanisms underlying the antioxidant and anti-inflammatory effects have been suggested to be due to the ability of EGCG to act as a scavenger molecule through enhancing the effect of enzymatic antioxidant defense mechanisms [18,19]. It has also been proven that EGCG can improve cell viability and has antiapoptotic properties, one of the most important antioxidant enzymes involved in these effects being represented by catalase (CAT) [20]. Another antiapoptotic mechanism acts by interfering with the NF-kB activation pathway [21]. The EGCG effect is related not only to inhibition of the nuclear factor KB (NF-kB), but also to mitogen-activated protein kinase (MAPK) pathways [22].

EGCG treatment decreases hepatic lipid peroxides and carbonyl formation, reducing oxidative stress [20]. Antioxidant enzymes increases after administration of EGCG in rats [14,23]. EGCG administration can regulate cell apoptosis by interfering with oxidative stress mechanisms (the most important mechanism revealed by experimental studies in vivo was represented by the ability to induce endogenous antioxidant systems) [13,24]. EGCG can reduce the production of several pro-inflammatory molecules such as tumor necrosis factor-alpha (TNF-alpha), monocyte chemoattractant protein 1 (MCP-1), intracellular adhesion molecule 1 (ICAM-1), nitric oxide (NO), regulated upon activation normal T cell expressed and presumably secreted (RANTES) molecules, vascular endothelial growth factor (VEGF), and matrix metalloproteinases (MMP) (important zinc-dependent enzymes responsible for extracellular matrix degradation) [19].

Our study aimed to evaluate if EGCG administration can reduce the hepatoxicity induced by experimental administration of gentamicin and investigate if the nano formulation of EGCG (liposomal formula) can improve its effects.

## 2. Materials and Methods

The experimental protocol was approved by the Ethics Committee of the Iuliu Hațieganu University of Pharmacy, Cluj-Napoca, Romania (no. 175/18.07.2019), and was conducted according to the rules of the European Convention for the Protection of Vertebrate Animals used for Experimental and Other Scientific Purposes.

### 2.1. Experimental Design

Thirty-five Wistar–Bratislava male rats, randomly distributed in five groups of seven animals, were procured from the Animal Department of the Iuliu Hațieganu University of Medicine and Pharmacy, Cluj-Napoca. All animals were kept in separate polypropylene cages in constant environmental conditions (24 ± 2 °C) and 60 ± 5% humidity, 12/12 h light/dark cycle. The animals weighing 200–250 g had unrestricted access to food (standard pellets from Cantacuzino Institute, Bucharest, Romania) and water. We applied 12 h of fasting before the blood sample collection.

Five groups of animals were used in our experiment as follows:

- control group (C): intraperitoneal (i.p.) administration of 1 mL saline solution 0.9%, daily for 7 days;
- G group: gentamicin (G) administration (1 mL i.p., 80 mg/kg b.w./day) daily for 7 days;
- G-EGCG group: gentamicin administration (1 mL i.p., 80 mg/kg b.w./day, unique dose) daily for 7 days, plus EGCG (1 mL i.p.) in a dose of 2.5 mg/0.1 kg b.w. 30 min before gentamicin administration, daily for 7 days;
- G-LEGCG group: gentamicin administration (1 mL i.p) daily for 7 days, unique dose plus liposomal EGCG (LEGCG) (1 mL i.p.), 2.5 mg/0.1 kg b.w. 30 min before gentamicin administration, daily for 7 days;
- G-Sily group: gentamicin + silymarin (100 mg/kg b.w.). Silymarin was administered i.p. once per day for 7 consecutive days, each day, 30 min before gentamicin administration [25].

All the chemicals (including EGCG and silymarin) were purchased from Sigma–Aldrich Co. (St. Louis, MO, USA). All other chemicals were of analytical grade.

### 2.2. Preparation and Physicochemical Characterization of EGCG-Loaded Liposomes

EGCG-loaded liposomes were prepared using 70 mM phospholipids (66.5 mM DPPC and 3.5 mM MPEG-2000-DSPE), and a 5:1 phospholipids:cholesterol molar ratio. The preparation protocol followed was as previously described [26]. Briefly, all the components of the lipidic film were dissolved in ethanol in a round-bottomed glass flask, the solvent was removed under reduced pressure and the obtained lipid film was hydrated with an aqueous EGCG solution (pH = 5.00), at 45 °C. An extrusion step followed, during which the liposomal dispersion was homogenized through polycarbonate membranes with a 200 nm final pore diameter, using LiposoFastLF-50 equipment (Avestin Europe GmbH, Mannheim, Germany). Finally, the unencapsulated EGCG fraction was removed through dialysis, using Slide-A-Lyzer filters (cassettes) with 10 kDa molecular weight cut-off.

The EGCG-loaded liposomes were characterized in terms of EGCG concentration, size, polydispersity index, and zeta potential. The liposomal EGCG concentration, as determined by UV-VIS spectrometry (Specord 200 Plus spectrophotometer, Analytik Jena, Überlingen, Germany) after the reaction with Folin–Ciocâlteu reagent, was around 900 μg/mL. The mean size and polydispersity index, assessed by a dynamic light scattering method (Zetasizer Nano ZS analyzer, Malvern Instruments Co., Malvern, UK), were 170 nm and less than 0.2, respectively. The zeta potential, measured by laser Doppler electrophoresis, was below −50 mV, ensuring good aggregative stability.

### 2.3. Blood Samples Collection and Measurement of Serum Markers

Blood samples were collected at the end of the experiment from the retro-orbital plexus of each animal under ketamine anesthesia (5 mg/kg b.w., i.p.) [27]. At the end of the experiment, the animals were euthanized by ketamine overdose administrated by intramuscular (i.m.) route. After serum separation (by blood centrifugation at 1500× *g* for 10 min), the following serum biomarkers were measured: urea, creatinine, basal glycemia, transaminases (aspartate aminotransferase-AST and alanine aminotransferase-ALT), oxidative stress/antioxidant balance, TNF-α, MMP-2, and MMP-9. The pancreatic metabolic function was reflected by basal glycemia (after 12 h of fasting). Transaminase assessment reflected the hepatocytolitic process consequent to gentamicin administration and was measured by an automated technique (Vita Lab Flexor E, Spankeren, The Netherlands). Urea and creatinine values were the parameters used for renal function evaluation after the toxicity of gentamicin administration. The oxidative stress parameters were measured according to Tsikas [28] for indirect NO (NOx) and Aebi [29] for catalase assessment [29,30]. Biochemical determinations were made by Spectroscopic measurements using a Jasco V-350 UV-VIS spectrophotometer (Jasco International Co., Ltd., Tokyo, Japan). The ELISA method was used (kit purchased from Signosis Inc., Santa Clara, CA, USA) for pro-inflammatory cytokine (TNF-α). Matrix metalloproteinase (MMP-2 and -9) measurements were also made by the ELISA method according to the manufacturer’s protocol (kit purchased from Elabscience Biotechnology Inc., Houston, TX, USA).

TNF-α, C-peptide, MMP-2, and MMP-9 measurements were made with kits purchased from R&D Systems Quantikine (McKinley Place NE, Minneapolis, MN, USA). All other chemicals were of analytical grade.

### 2.4. Histopathological Analysis

Each study animal’s liver slices were collected and fixed by immersion in Stieve solution for 24 h. After fixation, the fragments were dehydrated with ethyl alcohol, clarified with n-butanol, and embedded in paraffin. Sections with a thickness of 5 µm were made from the paraffin blocks. The sections were spread on slides, stained by the Goldner’s trichrome method, and examined with an optical microscope (Olympus BX41). Photographs were taken with an Olympus SC 180/cell Sens Entry 3.1.

### 2.5. Statistical Analysis

Serum marker data were described for each group as median (Q1 to Q3), arithmetic mean (SD) and (min to max) range (Q1 is the 25th percentile, Q3 is the 75th percentile, SD is the standard deviation, min is the minimum value and max is the maximum value). The evaluated serum markers were compared between groups as follow: C vs. G to show the inducement of toxicity, G vs. G-EGCG/G-LEGCG/G-Sily to show the effect of EGCG/LEGCG/Sily, G-EGCG vs. G-LEGCG to evaluate the differences between the used formula for EGCG, and G-EGCG/G-LEGCG vs. G-Sily to assess the effect of used LEGCG as compared to silymarin. The comparison between two groups was performed with the Mann–Whitney test. Data were analyzed with Statistica software (v. 13, StatSoft, Tulsa, OK, USA). The applied statistical tests were two-sided at a significance level of 0.05.

## 3. Results

Statistical analysis was conducted on all animals in each group since no animal died during the experiment.

### 3.1. Biochemical Markers

Gentamicin administration deteriorates the hepatic function evaluated with AST and ALT (Table 1). The administration of EGCG, LEGCG and silymarin decreases the damage of gentamicin on hepatic function with best results attributed to silymarin (Table 1). The used medication did not prevent the hepatic function damage expressed as the absence of significant differences between the control group and groups with medication (*p*-values < 0.0001).

Administration of gentamicin induces renal dysfunction, evaluated by creatinine, urea, and BUN (Table 2). Administration of liposomal EGCG showed better results than EGCG, however, silymarin showed significantly better efficacy (Table 2). The values of serum renal function markers on the group treated with silymarin were closest to the control group, but the differences are statistically significant (*p*-values < 0.0001).

Gentamicin administration impaired pancreatic function as indicated by significantly altering basal glycemia and C-peptide (Table 3). Liposomal EGCG proved significantly more effective than EGCG and showed similar efficacy as silymarin on basal glycemia (Table 3). None of the investigated drugs reached the basal glycemia, or C-peptide, as observed in the control group (*p*-values < 0.0001).

**Figure 1:**
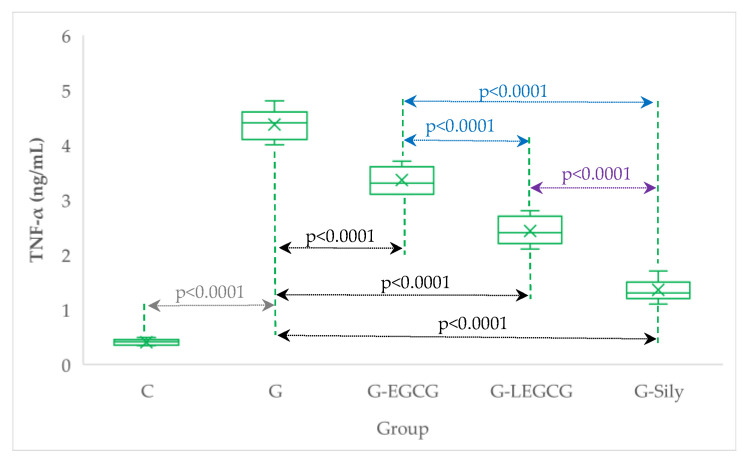
Serum levels of TNF-α by groups. C, control group; G, gentamicin group; G-EGCG, gentamicin and epigallocatechin gallate group; G-LEGCG, gentamicin and liposomal epigallocatechin gallate group; G-Sily, gentamicin and silymarin group.

**Figure 2:**
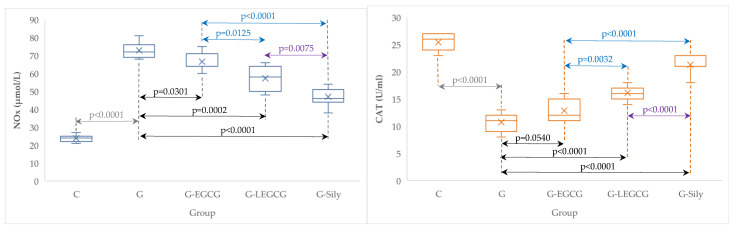
Serum levels of NOx and CAT by groups. C, control group; G, gentamicin group; G-EGCG, gentamicin and epigallocatechin gallate group; G-LEGCG, gentamicin and liposomal epigallocatechin gallate group; G-Sily, gentamicin and silymarin group.

Liposomal epigallocatechin gallate exhibits better effects on inflammation and oxidative stress/antioxidant balance than epigallocatechin gallate (Table 4, Figure 1 and Figure 2).

### 3.2. Matrix Metalloproteinase-2 and 9

**Figure 3:**
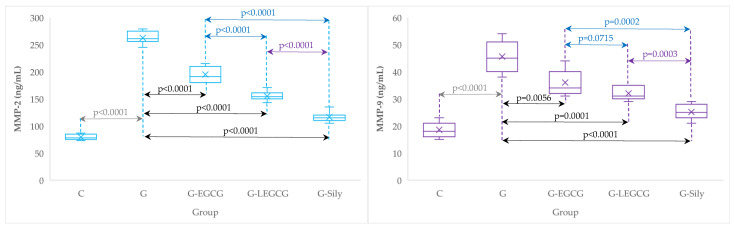
Serum levels of matrix metalloproteinases (MMP) 2 and 9 by groups. C, control group; G, gentamicin group; G-EGCG, gentamicin and epigallocatechin gallate group; G-LEGCG, gentamicin and liposomal epigallocatechin gallate group; G-Sily, gentamicin and silymarin group.

Gentamicin administration induced a significant increase in the serum value of both evaluated matrix metalloproteinases (control group compared to gentamicin group, Table 5, Figure 3). Silymarin had a better result in decreasing the serum value of matrix metalloproteinases compared to EGCG and liposomal EGCG (Table 5). EGCG and liposomal EGCG showed similar efficacy on serum values of MMP-9 (*p*-value > 0.05). The investigated drugs failed to restore the serum value of evaluated MMPs relative to the control group (*p*-value < 0.0001).

### 3.3. Liver Histopathology

**Figure 4:**
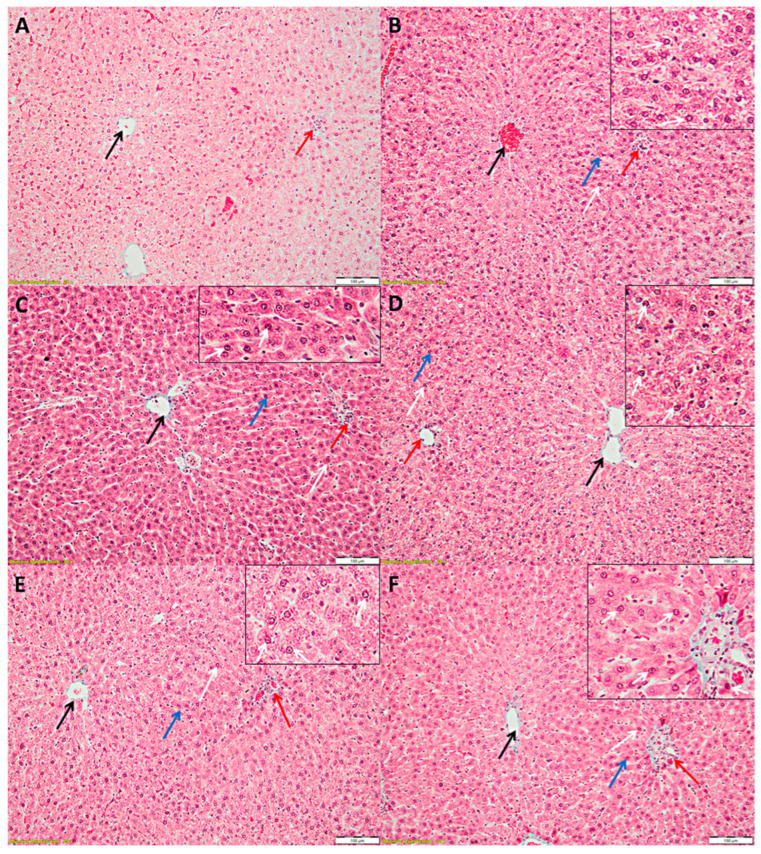
Hepatic lobes; (**A**)—Control (C) group; (**B**,**C**)—gentamicin (G) group; (**D**)—G-EGCG group; (**E**)—G-LEGCG group; (**F**)—G-Sily group, where EGCG = epigallocatechin gallate, LEGCG = liposomal epigallocatechin gallate, Sily = silymarin. The images were taken with a 20× lens and the scale bar is 100 µm. Black arrow—centrilobular vein; red arrow—porto-biliary space; blue arrow—hepatocytes with intensely stained cytoplasm; white arrow—thickened nuclear membrane.

The cytoarchitectonics of the liver was normal in the control (C) group. A typical appearance of hepatocytes arranged in the form of cords that converge from the periphery of the lobe to the centrilobular venule was observed in the C group (Figure 4A).

Most hepatocytes in the outer third of the hepatic lobe had a more intensely stained cytoplasm, while in the middle third, about half of the hepatocytes were more intensely stained in the G group. The number of hepatocytes with a slightly thickened nuclear membrane was lower in the G group (Figure 4B,C; gentamicin group).

In the case of the treatment with EGCG (G-EGCG group), most hepatocytes in the outer third of the hepatic lobe had a more intensely stained cytoplasm, while in the middle third, about half of the hepatocytes were more intensely stained. Furthermore, the number of hepatocytes with a slightly thickened nuclear membrane was lower than in the G group (Figure 4D).

Most of the hepatocytes in the outer half of the liver lobe in the rats treated with LEGCG (G-LEGCG group) appeared more intensely colored than those in the inner half, but the difference in intensity was not as large as in the G and G-EGCG group. Also, the number of nuclei thickened nuclear membrane was smaller than in the G or G-EGCG group (Figure 4E).

Most hepatocytes had a normal appearance in the G-Sily group. Only a few hepatocytes from the outer third of the liver lobes appeared with more intensely stained cytoplasm, and some hepatocytes had a slightly thickened nuclear membrane (Figure 4F).

No inflammatory aspects were observed in any of the groups. The only changes observed in the hepatocytes of animals in groups with gentamicin regardless of the added treatment were represented by a slight thickening of the nuclear membrane and more intense staining of the cytoplasm. More intense staining of the cytoplasm can be attributed to disruption of cellular metabolism with an aggregation of organelles in the cytoplasm.

## 4. Discussion

### 4.1. The Effects of EGCG and LEGCG on Hepatic Cells Function

The level of the transaminases in our study groups was proven to increase after gentamicin administration, with significant improvements in treated groups (silymarin, EGCG or LEGCG). The comparison of EGCG treatment with LEGCG showed significantly better results for the LEGCG molecule when compared with EGCG, but silymarin remained the most effective treatment (Table 1).

The effect of gentamicin on hepatic tissue was also proven by histopathological examination, LEGCG being similar to silymarin treatment. The beneficial effect of LEGCG could be based on the capacity of the liposomal formulation to have a better biodisponibility to the hepatic cells and to attenuate the toxicity of gentamicin. The lesional effect of gentamicin on hepatic cells, with transaminases elevation into the blood, is related to membrane lipids peroxidation, mitochondrial dysfunction and damage, oxidative/nitrosative stress enhancement, and finally hepatocyte destruction by apoptosis or necrosis [31]. It has been suggested that gentamicin proteotoxicity induces renal cell injury. Proteotoxicity results from an imbalance between misfolded protein accumulation due to translational errors and oxidative stress that exceed the ability of chaperones for protein refolding and repair [32,33]. Other nutraceuticals such as curcumin, cinnamic acid, honey, and propolis have been proven to significantly reduce gentamicin toxic effects [31,34,35]. All the beneficial effects demonstrated by the above-mentioned nutraceutical treatments have, as a pathophysiological loop, oxidative-nitrosative stress/antioxidant balance regulation [31,34,35]. Indeed, our study proved an improvement in nitrosative stress and antioxidant status after each of the treatments were administered which could explain the improvement of the hepatocyte stability using a similar mechanism. Dietary inclusion of *Allium sativum* (garlic) can also improve gentamicin-induced hepatotoxicity in rats, concurrently improving serum oxidative stress parameters induced by gentamicin administration [36]. Prevention of hepatic and renal toxicity induced by gentamicin was also demonstrated by the administration of snake venom extracted molecules (bradikinin-potentiating factor, BPF). These molecules have scavenging properties for oxidative molecules and anti-inflammatory effects [37]. Our study also demonstrated that gentamicin-induced liver histological alterations were successfully amended by EGCG and LEGCG treatments. Thickening of the nuclear membrane and more intense staining of the cytoplasm are early aspects encountered and described in cell death. These changes in the liver of the animals in groups that received gentamicin, at this stage, were reversible and did not necessarily lead to cell death. There were differences in the number of hepatocytes that suffered from one group to another, the most affected being the G group. Their number gradually decreased in the G-EGCG, G-LEGCG, and G-Sily group, so that in the G-Sily group, only a few hepatocytes were affected (Figure 4). Microscopic changes on hepatic tissue can also be improved by long term consumption of EGCG (in healthy Wistar rats) by attenuating the aging process [38]. One of the suggested mechanisms that contribute to the hepatoprotective effects is inducing the Nrf2 dissociation and its nuclear translocation [39]. Nrf2, in turn, can activate several antioxidant enzymes, such as heme-oxygenase-1 (HO-1), quinine oxidoreductase 1 (NQO1), and glutathione S-transferase (GST) [40]. However, the increase of ALT and AST in gentamicin treated rats implies the damage of liver cytoarchitecture and hepatic cell integrity, linked to microsomal membrane fluidity, mitochondrial dysfunction, and free radical generation. Impeded blood flow in liver tissue is an important consequence of injury augmentation and leakage of the transaminases into the bloodstream [41]. The mitochondria have a pivotal role in hepatic cells (designed to maintain an adequate metabolism for carbohydrates, lipids, and proteins). Therefore, hepatoprotective therapies must be able to maintain mitochondrial function within physiological parameters [42].

### 4.2. The Effects of EGCG and LEGCG on Renal Function

Improvement in renal function was significant after all treatments (EGCG, LEGCG, and silymarin) which was demonstrated by renal function parameter evaluation (urea, creatinine, and BUN) (Table 2). Gentamicin produced significant renal dysfunction as indicated by increased blood creatinine levels. The urea evaluation (resulting from protein metabolism) showed similar results (Table 2). Although the estimation of glomerular filtration rate (GFR) is a more complex parameter used to accurately indicate renal dysfunction, the creatinine biomarker usually along with urea measurement, is the most used parameter in experimental studies and clinical practice for renal dysfunction evaluation [43]. BUN (the nitrogen content of urea) showed similar results after EGCG, LEGCG, and silymarin administration. Over all, renal function biomarkers (creatinine, urea, and BUN) proved to be significantly improved after all treatments, the greatest effect being observed in the silymarin treatment group. Comparison of EGCG administration with the liposomal formula (LEGCG) showed superior efficacy for liposomal formulation. Nanoparticles charged with EGCG exerted a better protective effect on renal function due to their superior bioavailability for renal tissue. The mechanism of renal function protection is explained by improving the oxidative stress/antioxidant balance, reducing inflammation and metalloproteinase 2, and -9 levels [2]. EGCG was also shown to improve glomerular filtration rate through its capacity to activate the Nrf2 signaling pathway at different levels, e.g., by boosting the nuclear Nrf2 (the main regulator of antioxidant defense systems) through disrupting the interaction of Nrf2 with other molecules [44]. Increasing the Nrf2 level seems to be an important effect of epigallocatechin gallate (EGCG), increasing its protective role through modulation of Keap-1, HO-1, NQO-1, GPx, GCLc, GCLm, NF-kB cross-link, kinases, and apoptotic proteins [45].

### 4.3. The Effects of EGCG and LEGCG on Pancreatic Function

The liver represents one of the most important tissues for maintaining blood glucose in a normal range. The liver is where multiple metabolic pathways contribute to glucose homeostasis in feeding and fasting conditions by releasing or storing glucose molecules according to the need. The essential mechanisms activated to avoid hypoglycaemia are gluconeogenesis and glycogenolysis, and subsequently, glucose release from hepatocytes [46]. Postprandial, insulin prevents hyperglycemia, suppressing hepatic glycogenolysis and gluconeogenesis [39]. Liver tissue is one of the most sensitive tissues to oxidative stress, and function impairment can result from gentamicin administration [47]. Pancreatic tissue is also affected by the toxic effect of gentamicin, resulting in impaired insulin secretion (proven by significant changes in C-peptide) (Table 3). The C-peptide level can critically contribute to hyperglycemia induced by gentamicin administration (Table 3). C-peptide is the best measure of insulin secretion by pancreatic beta cells, being produced in equal amounts with insulin [48]. Insulin secreting beta cells (designed to compensate hyperglycemia) were incompetent in our study groups, resulting in low levels of C-peptide and hyperglycemia (Table 3). Still, compared with hyperglycemia resulting from Streptozotocin administration [49,50], the level of basal glycemia was lower. On the other hand, C-peptide can have a protective role in the histopathologic architecture of the liver. C-peptide administration prevented hepatic dysfunction associated with type 1 diabetes mellitus (DM). Therefore, C-peptide administration can be used as an alternative therapy for hepatocellular dysfunction in type 1 DM [51]. Moreover, experimental animal and human clinical studies have suggested a renoprotective effect of C-peptide when administrated in type 1 DM. The mechanism contributing to renoprotection exerted by C-peptide is based on reducing glomerular hyperfiltration and microalbuminuria [52].

### 4.4. The Effects of EGCG and LEGCG on Nitro-Oxidative Stress/Antioxidant Balance and Inflammation

Nitro-oxidative stress/antioxidant imbalance is one the most important pathogenetic mechanisms involved in tissue lesions associated with pro-inflammatory molecules contributing directly or indirectly to cell apoptosis and necrosis. Deciphering all the molecules contributing to tissue lesions seems to be a difficult challenge. Still, every component of this inflammation activation and persistence network could provide an important step for proper treatment. Since the contribution of nitric oxide (NO) in various and complex physiological processes and pathological mechanisms is considerable, this molecule serves as a standard for assessment of nitro-oxidative stress. NO mediates various deleterious pathways, which finally contribute to cell death. Our study showed a significant increase of NOx after gentamicin administration, proving the presence of nitro-oxidative stress due to gentamicin toxicity (Table 4, Figure 2). Improving the nitro-oxidative status in treated groups demonstrated the efficacy of these compounds, with LEGCG being the most effective (Table 4, Figure 2). The damage of cytoarchitectural hepatic integrity (proved by ALT and AST levels and histopathological examination) (Table 1 and Figure 4) is linked to oxidative/nitrosative stress, damage of hepatocytes, and transaminase release into the blood stream. NO molecules are highly reactive mediators released mostly by endothelial and Kuppfer cells of the liver to respond to various liver injuries [53]. Previous studies have shown that NOx significantly increases in gentamicin treated rats [2] and various nutraceutical molecules have proven to have beneficial effects [54,55,56]. Palm fruit extract also was proven to have protective effects against liver cytoarchitecture damage by gentamicin administration [57]. However, the mechanism that mitigates the gentamicin toxic effects on the liver and kidneys is due to its high content of phenolics and flavonoids [57]. Green tea phenolic compounds (particularly EGCG) exert antioxidant biologic activity acting over the precursor or directly on reactive oxygen and nitrogen species reducing oxidative/nitrooxidative undesirable reactions [58,59,60]. Remarkable improvement in NOx level and CAT (Table 4, Figure 2) correlated with significant reduction in blood transaminases (Table 1); and liver cytoarchitecture (Figure 4) was also obtained in our study after EGCG, LEGCG and silymarin administration. Compared with the control group, both EGCG and LEGCG treatments significantly improved nitro-oxidative stress/antioxidant balance, which was comparable with the silymarin effect. Silymarin (a complex mixture of polyphenolic molecules from *Silybum marianum*) had better results. Silymarin’s properties as a liver-protecting compound are traditionally used as an anti-inflammatory and anti-fibrotic treatment [9]. A precaution should be taken in patients with medication-treated diabetes mellitus and concomitant silymarin treatment due to its properties to reduce plasma glucose and glycated hemoglobin (hemoglobin A1c) levels. These patients risk hypoglycemia due to the potential additive effects of silymarin with antidiabetic medication [9].

Inflammation resulting from gentamicin administration had an injury effect on hepatic cells with cytosol release of transaminases, and ultimately severe cell dysfunction and death. Our results demonstrated a significant increase in TNF-alfa as a potential cytotoxic pro-inflammatory molecule that has been proven to contribute to liver damage substantially. Gentamicin-induced liver injury and an increase in serum AST, ALT, creatinine, BUN, and TNF-α together with concomitant histopathologic injury has already been proven to be related to increasing oxidative/nitrosative stress, decreasing antioxidant systems, and consequent inflammation, which leads to tissue injury [61]. EGCG and LEGCG had an anti-inflammatory effect in our study by significantly reducing the TNF-α level in both formulations, with better liposomal EGCG nano formulation results. The anti-inflammatory role of EGCG by reducing TNF-α level was also demonstrated by Hou et al., suggesting the mechanism is the influence on the NF-kb pathways, and through this mechanism, the suppression of production of a myriad of pro-inflammatory molecules, including TNF-α [62]. Again, silymarin demonstrated the best potency regarding the anti-inflammatory effect (Table 4, Figure 1) and consequently, the best hepatoprotective effect. Silymarin’s anti-inflammatory effects are related to the same mechanisms, one of them being represented by the ability of this compound to reduce TNF-α serum levels [63].

### 4.5. The Effects of EGCG and LEGCG on Serum Metalloproteinases

Significantly increased values of MMP-2 and -9, were observed after gentamicin administration (Table 5, Figure 3). Increased MMP production is related to excessive NOx generation due to nitro-oxidative stress, as we demonstrated in this experiment. NOx excess has a pathological vasodilatory effect and associated endothelial dysfunction [64]. The contribution of MMP to liver injury has already been demonstrated and was associated with oxidative/nitro-oxidative stress and inflammation [65]. MMP-9 is one of the most important metalloproteinases involved in inflammatory cell traffic associated with hepatic injury, and is expressed in activated leukocytes [66,67]. The exact role of MMP-2 in liver injury is still being researched, with recent reports indicating that MMP-2 contributes to leukocyte traffic in the hepatic circulation and to the anoikis process (a particular mechanism of cell death induced by cell detachment from the extracellular matrix) [68]. Degradation of the extracellular matrix component (EMC) by MMP contributes to a variety of liver diseases associated with hepatic fibrosis. The imbalance of extracellular matrix component turnover can also trigger inflammation, an immune response, and the onset and progression of liver fibrosis [69]. A reduction in MMP production has a beneficial effect on hepatic fibrosis, up-regulating these zinc-dependent enzymes has the ability to slow inflammation-dependent liver fibrosis [70]. Hepatocyte apoptosis is involved in liver fibrosis, the common trigger being increased oxidative/nitro-oxidative stress and the consequent inflammatory reaction. These processes ultimately lead to hepatocyte destruction and liver fibrosis onset (initiated by activated hepatic stellate cells) [71,72]. The liver injury induced by MMP activation is particularly complex and multifactorial. Therefore, further experimental studies are needed to determine the molecular mechanisms and identify better therapies to target specific pathophysiological mechanisms. This study demonstrated that LEGCG was the most effective at reducing the serum level of MMP-2 and -9, with the effect being similar to the silymarin effect (Table 5, Figure 3). However, regarding the downregulation of MMP-2 and -9, the comparison of the effect of LEGCG with silymarin, demonstrated a significant difference between these two formulations, with silymarin being better than LEGCG. In our study, amelioration of gentamicin injury effect through metalloproteinase modulation by EGCG (in both formula–solution and liposomal encapsulation) could be explained by the ability of EGCG to reduce contribution to the injury by both nitrosative stress and inflammation. To our knowledge, liposomal EGCG administration, as a hepatoprotector, in gentamicin induced liver injury was first described by this study. The comparative effect of LEGCG with the silymarin effect provides hope for a better therapy in the hepatoprotection therapeutic field (especially for patients with concomitant DM). Therefore, we consider that using EGCG in a nano formulation, as a liposomal particle charged with EGCG, in patients with DM, could have a better beneficial effect if both preparations are used (silymarin plus EGCG) as a combined therapy, or alone (LEGCG) in patients with DM. Further studies are needed to investigate this challenging hypothesis.

### 4.6. Potential Limitations and Further Studies

Although the experimental design was meticulous, several limitations of our study need to be discussed. First, serum concentrations of EGCG and LEGCG were not evaluated, so the association of circulating levels with the effect was not done. The process is elaborate and costly and the effort is worth it when a real benefit has been demonstrated. Second, the tissue concentrations of EGCG and LEGCG; and oxidative stress parameters, TNF-α, and MMPs were not measured. Quantifying these concentrations would better reflect the penetration at the tissue level, but the decision to measure them needs to be supported by reliable evidence. Third, short-term administration can not predict the long-term effect of EGCG or LEGCG on hepatic fibrosis progression. Fourth, Stieve mixture, which fixes the structures significantly better than formalin, limited the histopathology analysis. Immunohistochemical analysis is of interest as additional support for the reported results.

Understanding the hepatoprotective effects of EGCG and LEGCG would allow the establishment of effective therapeutic schemas. In this regard, apoptosis assessment by caspase 3 activity and cleaved-PARP expression levels would be a useful contribution. The results of our study support better hepatoprotective efficacy of LEGCG than EGCG and the association of LEGCG to silymarin is worth investigating. Furthermore, the histopathologic analysis of kidneys and pancreas in adding LEGCG to silymarin is under consideration for study by our team.

## 5. Conclusions

Liposomal epigallocatechin gallate exhibits superior hepatoprotective effects to EGCG, demonstrated by a better effect on reducing gentamicin-induced hepatotoxicity by reducing serum transaminases, nitric oxide, TNF-α, MMP-2, MMP-9, and improving catalase levels. Due to the higher beneficial effect of the nano formulation of EGCG, the liposome encapsulation of EGCG could be a potential candidate as adjuvant therapy for hepatic fibrosis onset and progression after a detailed evaluation of the pathophysiological mechanisms.

## Figures and Tables

**Table 1:** Hepatic function (serum markers—AST and ALT) after gentamicin administration. The comparative effect of EGCG, LEGCG, and silymarin.

Group	Statistics	AST (UI/L)	ALT (UI/L)
C	median (Q1 to Q3)	24.4 (23.4 to 24.8)	25 (24.3 to 26.3)
	mean (SD)	24.2 (1.2)	25.3 (1.6)
	(min to max)	(22.7 to 26.1)	(23.3 to 27.6)
G	median (Q1 to Q3)	165 (163.5 to 177.5)	207 (200 to 212.5)
	mean (SD)	169.4 (8.7)	205.6 (12.7)
	(min to max)	(159 to 180)	{185 to 222)
G-EGCG	median (Q1 to Q3)	134 (125.5 to 138.5)	157 (145 to 163.5)
	mean (SD)	130.9 (11.4)	156 (14.5)
	(min to max)	{111 to 143)	{138 to 180)
G-LEGCG	median (Q1 to Q3)	101 (98 to 106)	133 (123 to 146)
	mean (SD)	101 (7.8)	134.6 (13.2)
	(min to max)	{87 to 111)	{120 to 151)
G-Sily	median (Q1 to Q3)	79 (75 to 84)	115 (108 to 118)
	mean (SD)	79 (6.5)	113.3 (7.5)
	(min to max)	{69 to 87)	{103 to 123)
*p*-value	C vs. G	<0.0001	<0.0001
	G vs. G-EGCG	<0.0001	<0.0001
	G vs. G-LEGCG	<0.0001	<0.0001
	G vs. G-Sily	<0.0001	<0.0001
	G-EGCG vs. G-LEGCG	0.0001	0.0136
	G-EGCG vs. G-Sily	<0.0001	<0.0001
	G-LEGCG vs. G-Sily	0.0001	0.0030

AST, aspartate aminotransferase; ALT, alanine aminotransferase; Q1, 25th percentile; Q3, 75th percentile; SD, standard deviation; C, control group; G, gentamicin group; G-EGCG, gentamicin and epigallocatechin gallate group; G-LEGCG, gentamicin and liposomal epigallocatechin gallate group; G-Sily, gentamicin and silymarin group.

**Table 2:** Serum markers of renal function by groups after gentamicin administration. The comparative effect of EGCG, LEGCG, and silymarin.

Group	Statistics	Creatinine (mmol/L)	Urea (mmol/L)	BUN (mg/dL)
C	median (Q1 to Q3)	12 (10.5 to 13)	13.1 (11.8 to 13.8)	6.1 (5.5 to 6.4)
	mean (SD) (min to max)	12.1 (2.2) (10 to 16)	12.9 (1.4) (11.2 to 15)	6 (0.6) (5.2 to 7)
G	median (Q1 to Q3)	47 (46.5 to 48.5)	31.5 (30.7 to 32.8)	14.7 (14.3 to 15.3)
	mean (SD) (min to max)	47.6 (2) (45 to 51)	31.6 (1.8) (28.9 to 34)	14.7 (0.8) (13.5 to 15.8)
G-EGCG	median (Q1 to Q3)	40 (39 to 40.5)	25 (24.3 to 25.6)	11.7 (11.3 to 11.9)
	mean (SD) (min to max)	39.7 (1.1) (38 to 41)	24.9 (1.5) (22.5 to 27)	11.6 (0.7) (10.5 to 12.6)
G-LEGCG	median (Q1 to Q3)	30 (28 to 33)	21.7 (20.2 to 22.3)	10.1 (9.4 to 10.4)
	mean (SD) (min to max)	30.6 (3.2) (27 to 35)	21.2 (1.5) (19 to 23)	9.9 (0.7) (8.9 to 10.7)
G-Sily	median (Q1 to Q3)	25 (22 to 26)	15 (14 to 16.5)	7 (6.5 to 7.7)
	mean (SD) (min to max)	24.6 (3.2) (21 to 30)	15.1 (1.6) (13 to 17)	7.1 (0.7) (6.1 to 7.9)
*p*-value	C vs. G	<0.0001	<0.0001	<0.0001
	G vs. G-EGCG	<0.0001	<0.0001	<0.0001
	G vs. G-LEGCG	<0.0001	<0.0001	<0.0001
	G vs. G-Sily	<0.0001	<0.0001	<0.0001
	G-EGCG vs. G-LEGCG	<0.0001	0.0006	0.0006
	G-EGCG vs. G-Sily	<0.0001	<0.0001	<0.0001
	G-LEGCG vs. G-Sily	0.0042	<0.0001	<0.0001

BUN, Blood Urea Nitrogen; Q1, 25th percentile; Q3, 75th percentile; SD, standard deviation; C, control group; G, gentamicin group; G-EGCG, gentamicin and epigallocatechin gallate group; G-LEGCG, gentamicin and liposomal epigallocatechin gallate group; G-Sily, gentamicin and silymarin group.

**Table 3:** Impairment of pancreatic function induced by gentamicin by groups. The comparative effect of EGCG, LEGCG, and silymarin.

Group	Statistics	Basal Glycemia (mmol/L)	C-Peptide (pmol/L)
C	median (Q1 to Q3)	4.6 (4.5 to 4.7)	600 (585 to 612.5)
	mean (SD) (min to max)	4.6 (0.1) (4.5 to 4.7)	598.6 (17.5) (575 to 620)
G	median (Q1 to Q3)	8.8 (8.7 to 9.4)	363 (353 to 368)
	mean (SD) (min to max)	9 (0.7) (7.9 to 10)	360.7 (10.8) (345 to 375)
G-EGCG	median (Q1 to Q3)	7.2 (6.9 to 7.4)	390 (382.5 to 400)
	mean (SD) (min to max)	7.3 (0.4) (6.7 to 8.1)	391.4 (12.5) (375 to 410)
G-LEGCG	median (Q1 to Q3)	5.7 (5.5 to 6)	420 (412.5 to 425)
	mean (SD) (min to max)	5.7 (0.3) (5.3 to 6.1)	419.3 (10.2) (405 to 435)
G-Sily	median (Q1 to Q3)	5.2 (5.1 to 5.6)	460 (452.5 to 482.5)
	mean (SD) (min to max)	5.3 (0.3) (5 to 5.8)	465 (18.3) (440 to 485)
*p*-value	C vs. G	<0.0001	<0.0001
	G vs. G-EGCG	0.0001	0.0004
	G vs. G-LEGCG	<0.0001	<0.0001
	G vs. G-Sily	<0.0001	<0.0001
	G-EGCG vs. G-LEGCG	<0.0001	0.0006
	G-EGCG vs. G-Sily	<0.0001	<0.0001
	G-LEGCG vs. G-Sily	0.0646	0.0001

Q1, 25th percentile; Q3, 75th percentile; SD, standard deviation; C, control group; G, gentamicin group; G-EGCG, gentamicin and epigallocatechin gallate group; G-LEGCG, gentamicin and liposomal epigallocatechin gallate group; G-Sily, gentamicin and silymarin group.

**Table 4:** Serum markers of inflammation, oxidative stress/antioxidant balance by groups after gentamicin administration: comparative effect of EGCG, LEGCG, and silymarin.

Group	TNF-α (ng/mL)	NOx (µmol/L)	CAT (U/mL)
C	0.4 (0.1)	23.9 (2.0)	25.4 (1.5)
G	4.4 (0.3)	72.9 (4.5)	10.7 (1.8)
G-EGCG	3.4 (0.2)	66.6 (5.1)	12.9 (2.0)
G-LEGCG	2.4 (0.3)	57.3 (6.7)	16.1 (1.3)
G-Sily	1.4 (0.2)	47 (5.3)	21.3 (1.7)

Data are reported as mean (standard deviation); TNF-α, tumor necrosis factor alpha; NOx, nitric oxide; CAT, catalase; SD, standard deviation; C, control group; G, gentamicin group; G-EGCG, gentamicin and epigallocatechin gallate group; G-LEGCG, gentamicin and liposomal epigallocatechin gallate group; G-Sily, gentamicin and silymarin group.

**Table 5:** Serum levels of evaluated matrix metalloproteinases (MMPs) by groups after gentamicin administration: comparative effect of EGCG, LEGCG, and silymarin.

Group	MMP-2 (ng/mL)	MMP-9 (ng/mL)
C	79.3 (5.3)	18.6 (2.9)
G	262.6 (11.5)	45.6 (5.8)
G-EGCG	195 (13.9)	36 (4.8)
G-LEGCG	154.9 (9)	32 (2.4)
G-Sily	116.3 (9.6)	25.1 (2.8)

Data are reported as mean (standard deviation); MMP-2, matrix metalloproteinase 2; MMP-9, matrix metalloproteinase 9; C, control group; G, gentamicin group; G-EGCG, gentamicin and epigallocatechin gallate group; G-LEGCG, gentamicin and liposomal epigallocatechin gallate group; G-Sily, gentamicin and silymarin group.

## Data Availability

Data is contained within the article.
